# Functional Implication of Dp71 in Osmoregulation and Vascular Permeability of the Retina

**DOI:** 10.1371/journal.pone.0007329

**Published:** 2009-10-07

**Authors:** Abdoulaye Sene, Ramin Tadayoni, Thomas Pannicke, Antje Wurm, Brahim El Mathari, Romain Benard, Michel Joseph Roux, David Yaffe, Dominique Mornet, Andreas Reichenbach, Jose-Alain Sahel, Alvaro Rendon

**Affiliations:** 1 Institut National de la Sante et de la Recherche Médicale, UMR_S 968, Institut de la Vision, Paris, France; 2 Université Pierre et Marie Curie-Paris6, UMR_S 968, Paris, France; 3 Paul Flechsig Institute of Brain Research, Universität Leipzig, Leipzig, Germany; 4 Institut de Biologie Moléculaire et Cellulaire, Department of Neurobiology and Genetics, CNRS UMR 7104, Université de Strasbourg, Illkirch, France; 5 Department of Molecular Cell Biology, Weizmann Institute of Science, Rehovot, Israel; 6 Institut National de la Sante et de la Recherche Médicale, ERI 25 “Muscle et Pathologies”, Université Montpellier 1, EA 4202, CHU Arnaud de Villeneuve, Montpellier, France; 7 Centre Hospitalier National d'Ophtalmologie des quinze-vingts, Paris, France; Tufts University, United States of America

## Abstract

Functional alterations of Müller cells, the principal glia of the retina, are an early hallmark of most retina diseases and contribute to their further progression. The molecular mechanisms of these reactive Müller cell alterations, resulting in disturbed retinal homeostasis, remain largely unknown. Here we show that experimental detachment of mouse retina induces mislocation of the inwardly rectifying potassium channels (Kir4.1) and a downregulation of the water channel protein (AQP4) in Müller cells. These alterations are associated with a strong decrease of Dp71, a cytoskeleton protein responsible for the localization and the clustering of Kir4.1 and AQP4. Partial (in detached retinas) or total depletion of Dp71 in Müller cells (in Dp71-null mice) impairs the capability of volume regulation of Müller cells under osmotic stress. The abnormal swelling of Müller cells In Dp71-null mice involves the action of inflammatory mediators. Moreover, we investigated whether the alterations in Müller cells of Dp71-null mice may interfere with their regulatory effect on the blood-retina barrier. In the absence of Dp71, the retinal vascular permeability was increased as compared to the controls. Our results reveal that Dp71 is crucially implicated in the maintenance of potassium homeostasis, in transmembraneous water transport, and in the Müller cell-mediated regulation of retinal vascular permeability. Furthermore, our data provide novel insights into the mechanisms of retinal homeostasis provided by Müller cells under normal and pathological conditions.

## Introduction

Glial cells are crucially implicated in retinal function and integrity. Müller cells, the principal glia of the vertebrate retina are specialized radial glial cells which span the entire thickness of the retina and provide a wealth of functions that require an intimate interaction with the neurons and their synapses [1]. Like astrocytes in the brain, Müller cells in the retina are crucial for tissue homeostasis, particularly in deactivating and recycling neurotransmitters and in maintaining the ionic balance of the extracellular fluid [2]–[5]. Müller cells are also thought to participate in the induction, maintenance, and proper functioning of the blood-retina barrier (BRB) [6]–[8].

In the neural retina, potassium buffering and water drainage via Müller cells are mediated by the cooperation of inwardly rectifying potassium channels (Kir), especially Kir4.1, with the selective water transport protein, Aquaporin-4 (AQP4) [9], [10]. In healthy retina, Kir4.1 and AQP4 proteins are strongly expressed by Müller cells where these proteins are particularly enriched in the vitread endfeet and in cell processes abutting the intraretinal blood vessels [10], [11]. Proper functioning of Kir4.1 and AQP4 requires such a polarized expression of these channels in the plasma membrane of Müller cells [10]–[12]. The clustering and precise membrane localization of Kir4.1 and AQP4 are dependent on the dystrophin gene product, Dp71 [13]–[15]. In the normal retina, the Dp71 protein shows the same expression pattern as Kir4.1 and AQP4; it has been shown to be involved in the targeting of these channels to specific membrane microdomains via a macromolecular protein complex composed by Dp71 and dystrophin-associated proteins (DAPs) [14], [15]. Genetic inactivation of Dp71 (in Dp71-null mice) alters Kir4.1 and AQP4 distribution in Müller glial cells and this mislocation increases the vulnerability of retinal nerve cells to transient ischemia which is associated with neuronal cells death [14], [15]. Together, these two types of glial channels play a key role in K^+^ and water balance processes; noteworthy, their expression and/or cytotopographical distribution are altered in many instances of retinal injuries, including transient ischemia/reperfusion, diabetic retinopathy, retinal vein occlusion, and retinal detachment [14], [16]–[19]. Moreover, the early mislocation of Kir4.1 is accompanied by a dramatically decreased K^+^ conductance and a depolarization of the glial cell membrane which, in turn, impairs the function of the electrogenic uptake carriers for glutamate and GABA, subsequent neurotransmitter recycling, and other glia-neuron interactions [1], [20]. This may account for the occurrence of multiple and complex changes in neuronal and glial functions even before manifest vascular anomalies in diseases such as diabetic retinopathy can be detected [21]–[23], and suggests that the vascular pathology may be a consequence of disturbed glial cell functions.

Previous work on mice had shown that the deletion of Dp71 and the concomitant impairments in DAPs localization cause functio-morphological alterations of Müller glial cells, particularly in the endfoot region where Dp71 and DAPs are concentrated in wildtype retinas [15]. The purpose of the present study was to investigate the functional implication of Dp71 and the associated protein complex in the Müller cell-mediated maintenance of retinal integrity. For this purpose, we applied experimental retinal detachment as a well-established model of retinal injury [24]–[28] to both control (C57BL/6) and Dp71-null mice, and studied (i) the expression and cellular localization of the relevant proteins in Müller cells, (ii) the K^+^ currents across the Müller cell membrane, (iii) the swelling of Müller cell somata under osmotic challenge, and (iv) the permeability of retinal blood vessels. We found that experimental retinal detachment causes alterations in water transport and impairments in fluid absorption of Müller cells, associated with a downregulation of Dp71 expression. Similar functional deficits were observed in Müller cells from Dp71-null mice even without experimental retinal detachment; moreover, the permeability of the blood-retinal barrier was dramatically increased already in untreated Dp71-null mice as compared to the controls. These results support the view that Dp71 and the DAPs, by controling the expression and subcellular distribution of K^+^ and water channels, are essential for crucial glial cell functions such as retinal water homeostasis and maintenance of the BRB.

## Results

### Experimental Retinal Detachment induces reactive gliosis and alterations in protein expression in Müller cells

**Figure 1:**
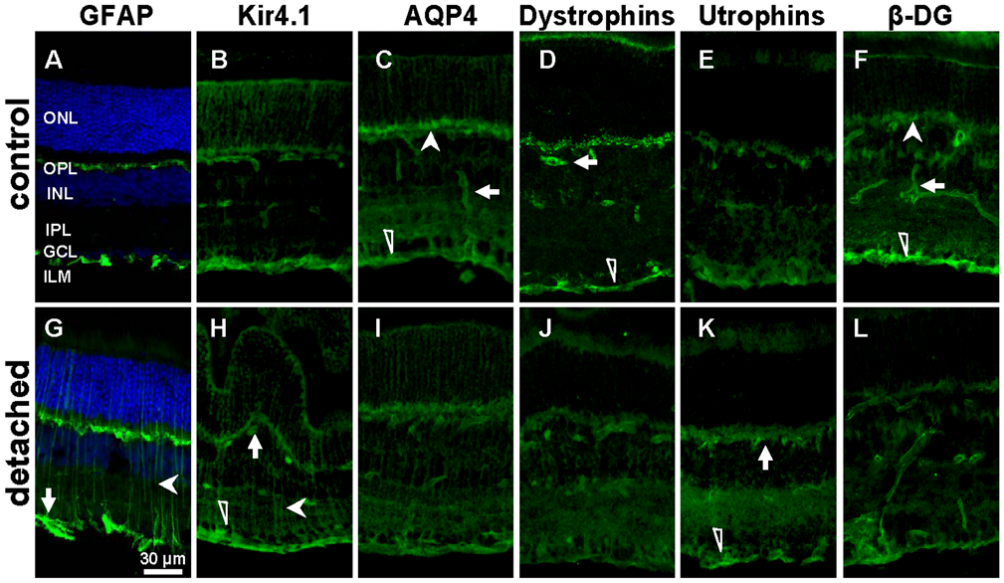
Experimental retinal detachment (RD) changed the expression and immunolocalization of GFAP, Kir4.1, AQP4, Dp71, Utrophin and β-dystroglycan. The slices were derived from control and detached retina of C57BL/6 mice 24 h after surgery. A,G: Retinal sections were probed with antibodies against GFAP (green) and for nuclei (Dapi, blue). Note the upregulation of GFAP after RD. B,H: Sections were also stained with antibodies against Kir4.1 (green). RD induced a mislocation of Kir4.1 along Müller cells (filled arrowhead) while the staining at the ILM (open arrowhead) and around blood vessels (arrow) remain unchanged. C,I: AQP4 staining (green) at the ILM (open arrowhead), around blood vessels (arrow) and at the OPL (filled arrowhead) was reduced in detached retina. D,J: Retinal sections were probed with a pan specific antibody against all forms of dystrophins (green). Dystrophin-Dp71 staining is localized at the ILM (open arrowhead) and around blood vessels (arrow), as previously reported [14] (Fig. S2). RD induced a reduction of Dp71 staining. E,K: 24 h after surgery Immunoreactivity for Utrophin (green) at the ILM (open arrowhead) was upregulated after RD. In control retina utrophin was localized at the ILM, around blood vessels and in the OPL. F,L: Sections were stained with antibodies against β-dystroglycan (β-DG). In control retina, β-DG is localized at the ILM (open arrowhead), around blood vessels (arrow) and at the OPL (filled arrowhead). RD induced a reduction of the staining in the ILM while the staining at the OPL (filled arrowhead) and around blood vessels (arrow) remain unchanged. ONL, outer nuclear layer; OPL, outer plexiform layer; INL, inner nuclear layer; IPL, inner plexiform layer; GCL, ganglion cell layer; ILM, inner limiting membrane. Scale bar = 30 µm.

**Figure 2:**
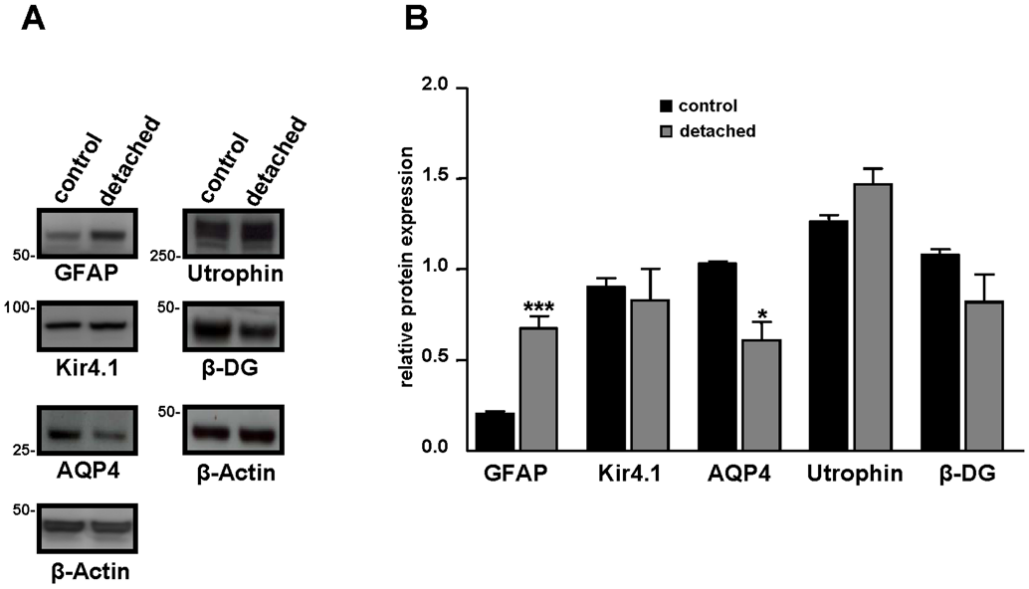
Relative retinal expression level of GFAP, Kir4.1, AQP4, Utrophin and β-dystroglycan after retinal detachment. A: 24 h after surgery, proteins of control and detached retina from C57BL/6 mice were extracted and Western blotting was performed. Representative photograph of immunoblots reacted with anti-GFAP, anti-Kir4.1, anti-AQP4, anti utrophins, anti β-dystroglycan (β-DG) and β-Actin antibodies. Numbers on the left refer to the relative electrophoretic mobility of prestained molecular mass standards in kiloDaltons. B: The relative protein level is expressed in arbitrary units. Each value represents the ratio of the specific band stain intensity normalized to β-Actin expression (TotalLab TL120, Nonlinear Inc, Durham NC, USA). In detached retina, GFAP expression level was significantly upregulated while AQP4 expression was downregulated. There was no significant difference in Kir4.1, utrophin and β-DG protein expression level after retinal detachment. All experiments were repeated at least three times, and the bars represent means + SE for triplicate data points; n = 4. *p<0.05; ***p<0.001 significant differences versus control.

To prove the induction of reactive Müller cell gliosis by experimental detachment, the expression of glial fibrillary acidic protein (GFAP) was assessed [29]. In control retinas, GFAP was only localized in astrocytes, at the inner retinal surface (Fig. 1A). Experimental retinal detachment caused an unequivocal upregulation of GFAP expression in Müller glial cells (Fig. 1G, arrowhead). Twenty-four hours after surgery, Western blot analysis confirmed an increased level of GFAP protein expression (Fig. 2A). For a semi-quantitative analysis, immunoblot band densities were quantified and normalized to β-Actin. This showed that in the detached retina, the GFAP level was elevated by a factor of 3 (Fig. 2B).

**Figure 3:**
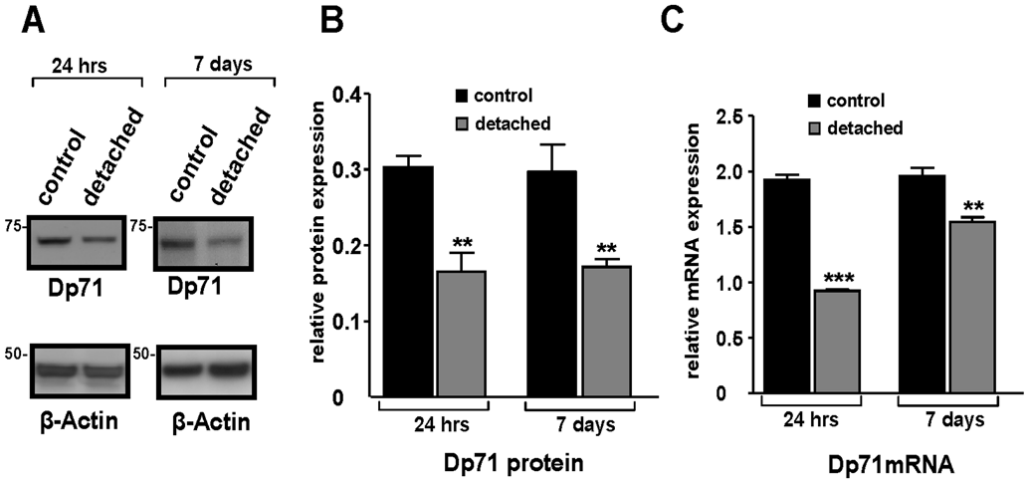
Dramatic reduction of Dystrophin Dp71 expression in detached retina. A: Western blot analysis of Dp71 and β-Actin total protein levels in control and detached retina 24 h and 7 days after experimental retinal detachment. Approximate molecular masses are given on the left of each panel in kiloDaltons. B: Dp71 and β-Actin expression were quantified by densitometry analysis of Western blots bands using TotalLab TL120 software (Nonlinear Inc, Durham NC, USA). The bar graph shows Dp71/β-Actin ratio. In detached retina, Dp71 protein level is dramatically reduced after 24 h and this decrease remains 7 days after surgery. C: The mRNA expression of Dp71 was determined by real-time PCR in control and detached retina at 24 h and 7 days after surgery and was normalized to the levels of mRNA for β-Actin. The Dp71 mRNA level in detached retina was strongly reduced. Data are expressed as mean + SE; n = 4. **p<0.01; ***p<0.001 significant differences versus control.

Immunohistochemistry demonstrated unequivocal Dp71 immunoreactivity mainly in the endfeet of Müller cells (Fig. 1D, open arrowhead) and around blood vessels (Fig. 1D, arrow) of control retinas, as previously reported [14] (Fig. S2) whereas immunolabeling was considerably reduced in detached retinas (Fig. 1J). To determine whether retinal detachment altered the protein and mRNA expression of Dp71 in C57BL/6 mice, we performed Western blot and real-time PCR analysis at 24 hours and 7 days after surgery. By using Western blotting and subsequent semiquantification of band densities of Dp71, we observed a dramatic reduction of Dp71 protein in detached retinas (Fig. 3A), by about 50% (Fig. 3B). Furthermore, we found that the Dp71 mRNA level in detached retinas was 50% lower than in control retinas, 24 hours after surgery. After 7 days, the Dp71 mRNA appeared to recover, as it was reduced by only 20% if compared to the controls. Together these data show that experimental retinal detachment-induced gliosis affects both gene and protein expression of Dp71 in Müller glial cells.

Kir4.1, the principal K^+^ channel in retina, was localized in the endfeet of Müller cells at the inner limiting membrane and around blood vessels of control retinas, as previously described [12]. In detached retinas, Kir4.1 was distributed all along the Müller cells fibers (Fig. 1H, filled arrowhead). However, Kir4.1 protein levels were not changed in detached retinas (Fig. 2B). Thus, retinal detachment induced a loss of the polarized expression of Kir4.1 but this mislocalization of Kir4.1 was not associated to a decrease of its expression level.

AQP4, the predominant aquaporin channel in retina, was found to be enriched at the endfeet of Müller cells (Fig. 1C, open arrowhead), around blood vessels (Fig. 1C, filled arrow) and in the outer plexiform layer (Fig. 1C, filled arrowhead), similar as previously reported [30]. At twenty-four hours of retinal detachment, the overall pattern of AQP4 distribution was unchanged but the immunolabeling intensity was decreased (Fig. 1I). This observation was confirmed by immunoblot analysis of AQP4 expression, revealing a significant decrease of the AQP4 expression level by 30% as compared to the controls (Fig. 2A,B).

Earlier it had been shown that utrophin, a cytoskeleton protein, shares the common expression pattern of Dp71, Kir4.1, and AQP4. Upregulation of utrophin may partially compensate Dp71 deficiency by interacting with AQP4 and Kir4.1 in retinas and isolated Müller cells of mice [14]. To further characterize utrophin localization and expression in detached retina, we performed immunohistochemistry and Western blot. In control retinas, utrophin was localized around blood vessels and in the inner limiting membrane (Fig. 1E). Retinal detachment induced an increase of utrophin immunolabeling in the inner limiting membrane (Fig. 1K, arrow and arrowhead). Although we observed an increase of utrophin staining, the slightly higher band density of Western blot was not significantly different (Fig. 2A,B).

As previously shown [14], the dystrophin-associated protein, β-dystroglycan (β-DG) is mainly localized in Müller cells endfeet (Fig. 1F, open arrowhead), around blood vessels (Fig. 1F, filled arrow) and in the outer plexiform layer (Fig. 1F, filled arrowhead). Immunolabeling at the inner limiting membrane was considerably reduced in detached retinas (Fig. 1L). Western blotting and subsequent semiquantification of band densities of β-DG revealed that the decrease of the band density of β-DG was not significantly different compared to control (Fig. 2A,B).

Summarizing these results, both gene and protein expression of Dp71 are strongly reduced after experimental retinal detachment in C57BL/6 mice. This may be responsible for the mislocation of Kir4.1 and the downregulation of AQP4, β-DG and syntrophins (Fig. S1) as well as for a compensatory upregulation of utrophin staining. To test this hypothesis, experimental retinal detachment was performed in the eyes of adult Dp71-null mice. As previously reported [14], [15], Kir4.1 and AQP4 were already mislocalized/downregulated in untreated Dp71-null mice, and retinal detachment failed to induce any additional alterations of these channels at 24 h or 7 days after surgery (data not shown). Furthermore, GFAP protein level was similar in untreated retinas of C57BL/6 and Dp71-null mice. Thus suggesting that Dp71 depletion did not induce reactive Müller cells gliosis.

### Retinal Detachment Causes Morphological and Physiological Alterations in C57BL/6 and Dp71-null mice

**Figure 4:**
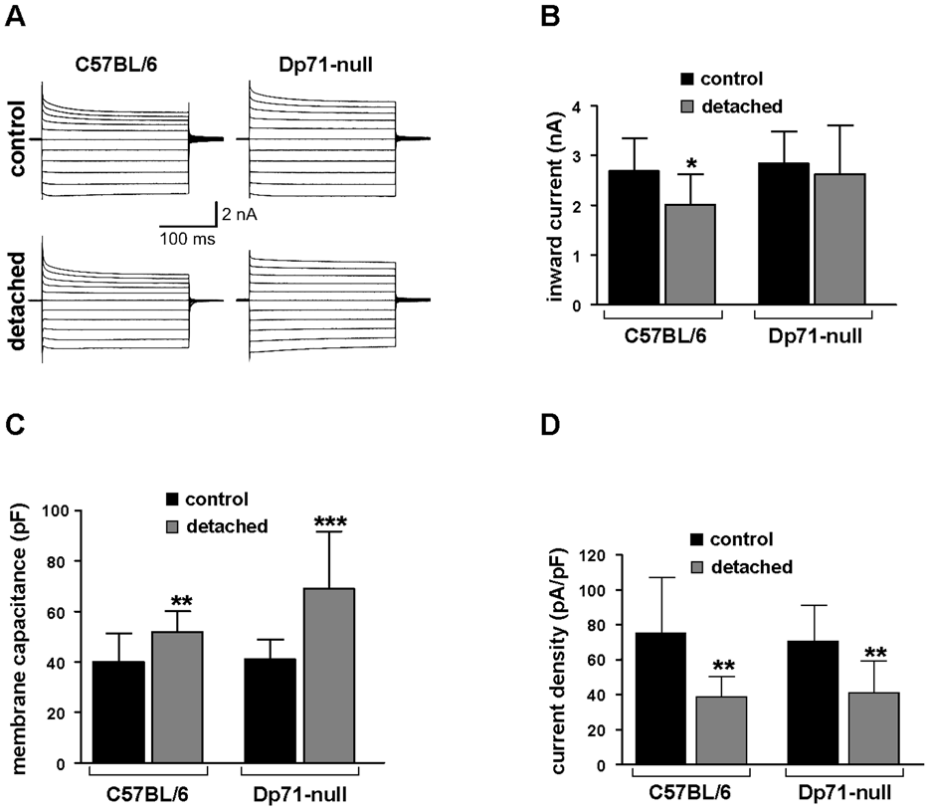
Effect of retinal detachment on electrophysiological properties of Müller cells. The cells were isolated from control and detached retina of wt and Dp71-null mice and were investigated 7 days after surgery. A: Examples of membrane currents evoked by depolarizing and hyperpolarizing voltage steps from a holding potential of −80 mV to potentials between −180 and +20 mV. No strong alterations of current kinetics were observed. B: Inward current amplitudes recorded at a −60 mV hyperpolarizing step. A weakly significant decrease was observed in cells from wt mice. C: Membrane capacitances were increased after retinal detachment in Müller cells from wt and Dp71-null mice. D: Resulting from decreased currents and increased capacitances, inward current densities were significantly reduced. Current densities were calculated from the data shown in B and C. Each bar represents mean values with standard deviations of 12 cells from 3 mice. Significant differences after retinal detachment versus the respective untreated control: * p<0.05, ** p<0.01, *** p<0.001.

Müller cells express the inwardly rectifying K^+^ channel Kir4.1 and it is well-known from other animal models that Kir-mediated currents may be markedly decreased under pathological conditions [16]–[18], [31]. Thus, we wanted to test whether retinal detachment of the murine retina could alter the K^+^ currents of Müller cells in a similar way. Electrophysiological properties (membrane currents, membrane potentials, membrane capacitances) were recorded in whole-cell patch-clamp experiments on isolated cells. Typical current patterns which could be recorded in voltage-clamp experiments are shown in Figure 4A. No remarkable differences in the current kinetics between both mouse strains and between control and detached retina were observed. For a more quantitative analysis, we recorded the inward currents evoked by a 60-mV hyperpolarizing step. As shown in Figure 4B, a weak reduction of inward current amplitudes was found, however, this decrease was not significant for Müller cells from detached retinal areas of Dp71-null mice. In C57BL/6 mice, a significant current decrease was observed although it was less prominent (by about 25%) than reported in rabbit and pig detachment models [27], [32]. The data suggest, that (i) the number of functional channels on the whole Müller cell is not different between wt and Dp71-null mice [14], and (ii) retinal detachment causes only weak, if any, functional inactivation of channels. Moreover, membrane capacitances were recorded after blocking the dominating K^+^ conductance by 1 mM Ba^2+^. The capacitance is a marker of the cell membrane area. There was no significant difference between the capacitance of Müller cells from untreated eyes of C57BL/6 mice as compared to cells from Dp71-null mice. However, Müller cells from the detached retina of the contralateral operated eye displayed a significant increase of their membrane capacitance in both strains (Fig. 4C), demonstrating cellular hypertrophy which is usually found in reactive Müller cells [16], [28], [32].

The slight decrease of inward current amplitude associated with the significant increase of the membrane capacitance resulted in a clear reduction of inward current densities (i.e., current amplitude divided by membrane capacitance) after retinal detachment (Fig. 4D). The current density is an electrophysiological parameter that describes the abundance of functional channels per membrane area. Its decrease could, in addition to channel mislocation (Fig. 1H), cause a restricted Müller cell function because extrusion of K^+^ ions into retinal blood vessels or into the vitreous in the process of spatial buffering should be impeded.

Other parameters of Müller cell electrophysiology (membrane potential, outward current amplitude, incidence of cells with fast voltage-dependent Na^+^ currents) did not show significant differences between control and detached retinas from both strains of mice.

### Osmotic Müller Cell Swelling of C57BL/6 injured and Dp71-null retinae

**Figure 5:**
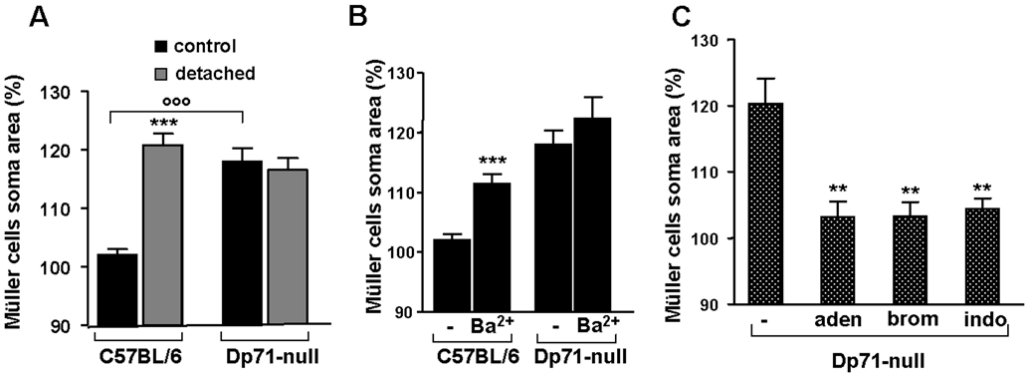
Osmotic swelling properties of Müller glial cells. Müller cell somata were measured after 4 minutes perfusion of retinal slices with a hypotonic solution (60% of control osmolarity). Data were expressed in percentage of the values obtained before hypotonic challenge. A: Müller cells of control and detached retina from wt and Dp71-null mice were exposed to hypotonic challenge. In wt mice, only cells of detached retina swell. Note the swelling of Müller cells from control retina of Dp71-null mice. B: Müller cells of control retina from wt and Dp71-null mice were exposed to hypotonic challenge in the presence of barium chloride (Ba^2+^). Addition of Ba^2+^ induced a swelling of Müller cells of wt mice and had no effect on the soma area of Müller cells of Dp71-null mice. C: Addition of adenosine (aden); inhibitor of phospholipase A_2_, 4-bromophenacyl bromide (brom) and inhibitor of cyclooxygenase enzyme, indomethacin (indo) blocked the swelling of Müller cells of control retina from Dp71-null mice. Each bar represents value obtained in 12–34 cells; n = 3 mice for each group. Data are expressed as mean + SE. **p<0.01; ***p<0.001 significant differences versus control. °°°p<0.001 significant difference versus wt.

Previous work has revealed that reactive Müller cells may display alterations in transmembraneous water transport [16]–[19]. Thus, the swelling of Müller cell somata was investigated in acutely isolated retinal slices by perfusing the slices with a hypotonic solution containing 60% of control osmolarity. Exposure to hypotonic solution did not alter the size of Müller cell bodies in retinal slices from untreated eyes of C57BL/6 mice (102.0±1.0%, n = 34). However, a 4-min perfusion of slices from detached retinae of this strain caused an increase in the size of Müller cell somata to 120.7±2.0% (n = 24, *P*<0.001) (Fig. 5A). Müller cell bodies in slices from control retinas swelled upon hypotonic stress when K^+^ channel-blocking Ba^2+^ ions were present in the bath solution (111.5±1.6%, n = 15; *P*<0.001). By contrast, Müller cells in retinal slices from untreated eyes of Dp71-null mice already displayed osmotic swelling to 118.0±2.3% (n = 27; P<0.001 as compared to C57BL/6 Müller cells under the same condition) in the absence of Ba^2+^. Müller cells in the detached retina of Dp71-null mice swelled to an extent that was not significantly different to that of the untreated eye (Fig. 5A; 116.6±2.0%, n = 26). Moreover, the application of Ba^2+^ failed to induce an additional swelling in Müller cells from untreated Dp71-null mice (Fig. 5B; 122.3±3.6%, n = 12).

As it had been shown in a pig model of retinal detachment that osmotic Müller cell swelling can be inhibited by the application of adenosine or of blockers of pathways that produce inflammatory mediators [19], the nucleoside adenosine (10 µM) was applied with the hypotonic solution onto untreated retinas from Dp71-null mice. Adenosine significantly blocked somatic swelling (103.2±2.2%, n = 12; in comparison to 120.3±3.8%, n = 8, without adenosine; *P*<0.01). The enzymes, phospholipase A_2_ and cyclooxygenase produce the inflammatory mediators arachidonic acid and prostaglandins, respectively. The selective inhibitors of phospholipase A_2_, 4-bromophenacyl bromide (300 µM), and of cyclooxygenase, indomethacin (10 µM), prevented the swelling of Müller cells from Dp71-null mice (Fig. 5C; 103.3±2.1%, n = 8, *P*<0.01 and 104.4±1.6%, n = 7, *P*<0.01, respectively). These data suggest that deletion of Dp71 modifies the osmotic swelling characteristics of Müller cells, and that inflammatory mediators are involved in this alteration.

### Dp71 Deletion in Müller Cells increases the BRB Permeability

**Figure 6:**
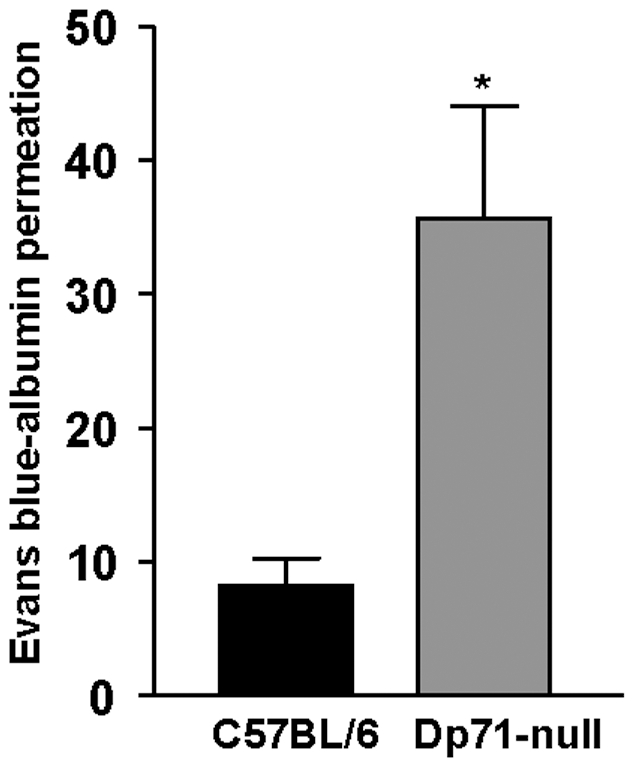
Implication of Dp71 in blood-retinal barrier permeability. Blood-retinal barrier permeability was measured using Evans blue dye vascular permeability assay on wt and Dp71-null adult mice. Vascular permeability was strongly increased in Dp71-null mice compared with C57BL/6 mice. Data are expressed as mean + SE; n = 4. *p<0.05; significant differences versus control.

There were two previous observations which prompted us to study the effect of Dp71 depletion on BRB integrity. First, Müller glial cells are in close contact with retinal blood vessels and play an important role in the formation and maintenance of the BRB [6], [8]. Second, Dp71 immunoreactivity is localized in Müller cell endfeet and processes surrounding retinal blood vessels [14] (Fig. S2). Retinal vascular permeability was assessed in C57BL/6 and Dp71-null adult mice using the Evans blue-dye technique [33]. This revealed that the BRB permeability of Dp71-null mice was up to 4-fold elevated (p<0.05) as compared to C57BL/6 mice (Fig. 6). This shows that the absence of Dp71 in Müller cells is accompanied by an increased permeability of the BRB, and may even be indicative of a causal relation between the two features.

## Discussion

Using an experimental mouse model of retinal detachment, we (i) confirm morpho-functional alterations in reactive Müller glial cells including a mislocation of Kir4.1 potassium channels and a downregulation of AQP4 water channels, accompanied by disturbed volume regulation of the cells; (ii) show a fast and strong decrease of the dystrophin protein, Dp71; and finally (iii) demonstrate an impaired BRB function as evidenced by increased retinal vascular permeability. These findings indicate a key role for glial Dp71 in the maintenance of retinal potassium and water homeostasis and cellular volume regulation, as well as in the control of the BRB.

In our model of experimental detachment in murine retinas, Müller cells became reactive (as indicated by GFAP expression, Fig. 1G) and showed a decrease in potassium currents, similar as found in rabbit and porcine models of retinal detachment [27], [32]. Here we show, for the first time, a fast decrease in gene and protein expression of Dp71 (Fig. 3). This may indicate that generally, the mislocation of Kir4.1 channels in reactive Müller cells results from an alteration in Dp71 expression. This hypothesis is supported by earlier data showing that Dp71 is required for proper clustering and precise membrane localization of Kir4.1 and AQP4 channels [13]–[15]. Most likely, the function of Dp71 is to stabilize the Kir4.1 and AQP4 channel molecules in specific membrane domains by limiting their lateral diffusion. Our present results confirm that functional expression of Dp71 is necessary for the highly asymmetric expression of the inwardly rectifying potassium channel Kir4.1 in Müller cells, and suggest that Dp71 downregulation is one step upstream of the Kir4.1 and AQP4 channel alterations in the signaling cascade of reactive gliosis.

This idea is further supported if the consequences of Dp71 deficiency are studied one step downstream of the channel alterations, by assessing the swelling properties of Müller cells. We observed a similar anomalous osmotic swelling in Müller cells from untreated Dp71-null mice and in cells from C57BL/6 after detachment-induced gliosis (Fig. 5). This finding shows that partial or total depletion of Dp71 in Müller cells are associated with alterations in transmembraneous water transport.

In addition to failures in Kir4.1 and AQP4 expression and/or membrane insertion, Dp71 deficiency may also induce a chronic inflammation. We found that similar as in a porcine model of retinal detachment [19], inflammatory mediators are implicated in osmotic Müller cell swelling in Dp71-null mice. Inhibition of phospholipase A_2_ or cyclooxygenase, enzymes producing arachidonic acid and prostaglandins, respectively, prevent the osmotic swelling of Müller cells from Dp71-null mice. We thus suggest that Müller cells of Dp71-null mice undergo a chronic inflammation which contributes to the alterations in their physiological parameters. Indeed, the gene expression of cyclooxygenase-2, known to play a key role in the pathogenesis of inflammation, was up to 7-fold elevated in retinas of Dp71-null mice at postnatal day 6 as compared to C57BL/6. However, there was no significant difference between the cyclooxygenase-2 level of retinae from C57BL/6 and those from Dp71-null adult mice (unpublished data). Further studies are necessary to elucidate the underlying pathomechanisms.

Extracellular accumulation of fluid, resulting in edema, is the most common cause of vision impairment in diabetic retinopathy [34]. Extravascular accumulation of fluid in the subretinal space and the inner retinal tissue is normally prevented by pigment epithelium and Müller cells [35]. Vascular leakage and impaired fluid absorption from the retinal tissue across the glio-vascular interface are major pathogenic events of edema formation [36]. We show here that both pathomechanisms are stimulated by the partial or total absence of Dp71. First, the fluid transport through Müller cell membrane is impaired (Fig. 5) and second, we show here for the first time that Dp71 depletion is accompanied by a significantly increased retinal vascular permeability (Fig. 6). This latter observation is in accordance with previous reports showing that Müller cells participate in the maintenance and the proper functioning of the BRB [6]–[8]. In the retina, Dp71 is mainly expressed in Müller cells endfeet and processes surrounding blood vessels. It is thus conceivable that the lack of Dp71 results in morphological and functional alterations of these vitread and perivascular Müller cell compartments and that these impairments lead to a dysregulation of retinal vascular permeability. Presently the underlying molecular signaling cascade of this putative pathomechanism is completely unclear. It may be noteworthy in this context that Müller cells are capable of secreting the vascular endothelial growth factor (VEGF) [37], [38], a key player in increasing the retinal vascular permeability. Obviously, the ‘compensatory’ upregulation of utrophin observed at the inner limiting membrane (Fig. 1K) is not functionally compensating the absence of Dp71. Under these circumstances, it appears to be surprising that Dp71-null mice do not show impairments in visual functions nor indications of retina degeneration if not challenged [14]. Very probably, the animals develop partial compensatory mechanisms; however, this remains to be proven by the future generation of conditional Dp71 KO mice.

In conclusion we show here that depletion of Dp71, a cytoskeleton protein associated to the membrane, leads to physiological alterations in Müller cells similar to those observed in injured or diseased retinas; this involves a mislocation of potassium and water channels (Kir4.1 and AQP4) and a consequent dysregulation of water transport through Müller cells. Furthermore, we show that the early, acute downregulation of Dp71 in normal animals may be an important, decisive step in the development of reactive gliosis, upstream of the physiological alterations. Finally we show the altered Müller cell properties in Dp71-null mice may increase the retinal vascular permeability, via a hitherto unknown mechanism. Taken together, Dp71-null mice appear as a versatile, promising model to develop novel drugs for the resolution of retinal edemas such as observed in human diabetic retinopathy.

## Materials and Methods

### Ethics Statement

All animal procedures were conducted in accord with UPMC University and INSERM Institutional Animal Care Committee guidelines. Surgery was carried out and/or directly supervised by persons with appropriate levels of experience and training, and surgery performed on animals that will survive was undertaken with careful attention to aseptic technique and prevention of infection. All surgical procedures were completed under anesthesia that renders the animal insensitive to pain.

### Animals

The Dp71-null mice [39] were obtained by replacing, via homologous recombination, most of the first and unique exon of Dp71 and of a small part of Dp71 first intron with a sequence encoding a β-gal-neomycine-resistance chimeric protein (β-geo). This abolished the expression of Dp71 without interfering with the expression of other products of the DMD (Duchenne Muscular Dystrophy) gene. C57BL/6J mice strain (Charles River, France) was used as controls for this study.

### Antibodies

Monoclonal antibodies targeting β-Actin and GFAP were purchased from Sigma-Aldrich (Deisenhofen, Germany) and monoclonal antibodies directed against β-dystroglycan (43 DAG) were purchased from Novocastra Laboratories (Newcastle-upon-Tyne, UK). Polyclonal antibodies directed against dystrophins (H4) and utrophin (K7) were previously characterized [40], whereas the ones directed against Kir4.1 and AQP4 were from Alomone Labs (Souffelweyersheim, France).

### Induction of retinal detachment

Local retinal detachment was induced in the right eye of adult mice, as previously reported with minor modification [41]. Briefly, mice were anesthetized by peritoneal injection of a mixture of 62.5 mg/kg ketamine (Virbac, France) and 12.5 mg/kg xylazine (Bayer, France). The pupils of the eyes were dilated by topical tropicamide 0.5% (Thea, France) and retinal detachment was created by subretinal injection of sodium hyaluronate. The left eye, sham injected, served as control. Animals with lens injury, vitreous hemorrhage and eye infection were excluded from this study.

### Immunohistochemistry

Enucleated eyes were dissected to remove lens and cornea, and fixed by immersion in 4% paraformaldehyde for 1 h. Fixed eyes were cryoprotected in 30% sucrose, frozen and embedded in Cryomatrix (Thermo Shandon, Pittsburgh, Pennsylvania, United States), and then cut into 10 µm cryostat sections and mounted on SuperFrost/Plus slides (O. Kindler, Freiburg, Germany). Sections were permeabilized for 10 minutes with 0.1% PBS (Phosphate Buffer Saline) -Triton X100 and blocked for 1 h with 0.1% normal goat serum, 3% bovine serum albumin, 0.1% Tween 20, and PBS. Sections were then incubated with primary antibody for 2 h at room temperature. After several washes with PBS, secondary antibodies (Interchim, France) coupled to Alexa fluor (Invitrogen, France) were used diluted 1:500 in 0.5% bovine serum albumin for 1 h. Sections were washed, mounted with Fluorsave reagent (Calbiochem, San Diego, CA, USA) and viewed with a DM 5000B microscope (Leica Microsystems SAS, Rueil Malmaison, France) equipped with a Photometrics cool SNAP TM FX camera (Ropper Scientific, Tuscon, AZ, USA).

### Western blot analysis

Western blot analysis was performed as previously described [42]. In brief, retinal protein extracts were resolved using NuPAGE Tris–Acetate 3–8% gradient gels (Invitrogen, France) and electrotransferred to polyvinylidene difluoride (PVDF) membranes according to the manufacturer's instructions. PVDF membranes were blocked in PBS containing 0.1% Tween 20, 1% BSA, 5% dry milk (BIO-RAD, Herts, UK) for 1 h at room temperature then incubated with the primary antibody in the same blocking buffer. Blots were then washed and incubated with the secondary antibody conjugated to horseradish peroxidase (Jackson Immunoresearch laboratories). Chemiluminescence was performed using ECL plus Western blotting detection system (GE Healthcare, Germany) and documented on film (GE Healthcare, Germany).

### Quantitative RT-PCR analysis of retinal RNA

Total RNA from retina was extracted using Trizol reagent (Invitrogen, France) according to the manufacturer's instructions. Reverse transcription was performed on 1 µg total RNA using SuperScript II and random hexamers (Invitrogen, France). PCR amplifications of cDNA were performed using Master plus SYBR Green I (Roche Diagnostics, Germany) on a LightCycler instrument (Roche Products, Basel, Switzerland). PCR primers were designed using Primer3 software [43]. The following primer pairs were used: Dp71, sense 5′-ACAACCATGAGGGAACACCT-3′, anti-sense 5′-TCTGGAGCCTTCTGAGCTTC-3′; β-Actin, sense 5′-GCTCTTTTCCAGCCTTCCTT-3′, anti-sense 5′-CTTCTGCATCCTGTCAGCAA-3′. For relative comparison, the C_t_ values of real-time PCR results were analysed using the ΔC_t_ method according to the manufacturer's instructions. The amount of Dp71 cDNA was normalized to the standard internal control obtained using primers for β-Actin.

### Glial cell isolation

The retinae were isolated and special care was taken to separate detached areas. Retinal pieces were incubated in papain (0.2 mg/ml; Roche Molecular Biochemicals, Mannheim, Germany)-containing Ca^2+^- and Mg^2+^-free phosphate-buffered saline, pH 7.4, for 30 minutes at 37°C, followed by several washing steps with saline. After short incubation in saline supplemented with DNase I (200 U/ml; Sigma, Taufkirchen, Germany) the tissue pieces were triturated by a pipette, to obtain suspensions of isolated cells. The cells were stored at 4 °C in serum-free minimum essential medium (Sigma) until use within three hours after isolation.

### Electrophysiological recordings

Membrane currents of acutely isolated glial cells were recorded in the whole-cell configuration of the patch-clamp technique. Voltage-clamp records were performed at room temperature (22–25°C) using the Axopatch 200A amplifier (Axon Instruments, Foster City, CA) and the ISO-2 computer program (MFK, Niedernhausen, Germany). The signals were low-pass filtered at 1, 2, or 6 kHz (eight-pole Bessel filter) and digitized at 5, 10, or 30 kHz, respectively, using a 12-bit A/D converter. Patch pipettes were pulled from borosilicate glass (Science Products, Hofheim, Germany) and had resistances between 4 and 6 MΩ when filled with a solution containing (mM) 10NaCl, 130 KCl, 1 CaCl_2_, 2 MgCl_2_, 10 ethyleneglycolbis(aminoethylether)tetra-acetate, and 10 *N*-2-hydroxyethyl-piperazine-N'-2-ethanesulphonic acid (HEPES) adjusted to pH 7.1 with Tris-base. The recording chamber was continuously perfused with extracellular solution which contained (mM) 135 NaCl, 3 KCl, 2 CaCl_2_, 1 MgCl_2_, 1 Na_2_HPO_4_, 10 HEPES, and 11 glucose; the solution was equilibrated to pH 7.4 with Tris-base.

To evoke membrane currents, de- and hyperpolarizing voltage steps of 250 ms duration, with increments of 10 mV, were applied from a holding potential of −80 mV. The amplitudes of the steady-state inward currents were measured at the end of the voltage step from −80 to −140 mV. To activate transient voltage-dependent currents, a hyperpolarizing prepulse (500 ms, −120 mV) was applied before depolarization. The membrane capacitance of the cells was measured by the integral of the uncompensated capacitive artefact (filtered at 6 kHz) evoked by a hyperpolarizing voltage step from −80 to −90 mV when Ba^2+^ ions (1 mM) were present in the bath solution to block the K^+^ conductance. Membrane potentials were measured in the current-clamp mode. Statistical analysis (unpaired Student's *t* test) was performed using the SigmaPlot program (Jandel Corp.); data are expressed as means±standard deviation. Cells from the operated and the contralateral control eyes from 3 C57BL/6 and 3 Dp71-null mice were used.

### Müller cell swelling

To determine volume changes of Müller glial cells evoked by hypotonic stress, the cross-sectional area of glial cell somata in the inner nuclear layer of retinal slices was measured. Acutely isolated slices (thickness, 1 mm) were prepared from untreated control eyes and from the detached retinal area of operated eyes from C57BL/6 and Dp71-null mice, placed in a perfusion chamber and loaded with the vital dye Mitotracker Orange (10 µM). This dye is selectively taken up by Müller glial cells whereas retinal neurons, astrocytes, and microglial cells remain unstained [44]. The stock solution of the dye was prepared in dimethylsulfoxide and resolved in saline. The slices were examined with a confocal laser scanning microscope LSM 510 Meta (Zeiss, Oberkochen, Germany). Mitotracker Orange was excited at 543 nm, and emission was recorded with a 560 nm long-pass filter. To assure that the maximum soma areas were precisely recorded, the focal plane was continuously adjusted in the course of the experiments.

A gravity-fed system with multiple reservoirs was used to perfuse the recording chamber continuously with extracellular solution; the hypotonic solution and test substances were added by rapid change of the perfusate. The extracellular solution consisted of (in mM) 136 NaCl, 3 KCl, 2 CaCl_2_, 1 MgCl_2_, 10 HEPES, and 11 glucose, adjusted to pH 7.4 with Tris-base. The hypotonic solution (60% of control osmolarity) was made up by adding distilled water. BaCl_2_ (1 mM) was preincubated for 10 minutes in extracellular solution before application within hypotonic solution. Blocking substances were preincubated for 15 to 45 minutes, and adenosine was administered simultaneously with the hypotonic solution. Swelling experiments were performed at room temperature.

To determine the extent of glial soma swelling, the cross-sectional area of Mitotracker Orange-stained cell bodies in the inner nuclear layer of retinal slices was measured off-line using the image analysis software of the LSM. Bar diagrams display the mean (+ SEM) cross-sectional area of cell somata that was measured after 4-minute perfusion with hypotonic solution, in percent of the soma area measured before osmotic challenge (100%). The absolute control values were not different between the several groups and varied in the range between 47 and 51 µm^2^.

### Quantification of blood-retinal barrier permeability

Vascular permeability was quantified by measuring albumin leakage from blood vessels into the retina using the Evans blue method [33]. Briefly, mice were anesthetized and Evans blue (45 mg/kg; Sigma-Aldrich, Germany) was injected through the jugular vein. Blood samples were taken 3 h after injection of the dye and mice were perfused for 2 minutes via the left ventricle with a citrate buffer (0.05 M, pH 3.5) prewarmed to 37°C. After perfusion, both eyes were enucleated and carefully dissected. Retinas were dried in a Speed-Vac for 5 h, weighed and the Evans blue dye was extracted by incubating the retina with 100 µl of formamide for 18 h at 70°C. Retina samples were centrifuged at 70,000 rpm for 20 minutes and blood samples were centrifuged at 12,000 rpm for 15 minutes. Both supernatants were used to measure absorbance. A background-subtracted absorbance was determined by measuring each sample at both 620 nm, the absorbance maximum for Evans blue, and 740 nm, the absorbance minimum. Evans blue concentration in the plasma and the retina was calculated from a standard curve of Evans blue in formamide. Blood-retinal barrier permeability was expressed in microliter of Evans blue per gram of wet retina per hour (µl Evans blue × g wet retina^−1^ × h^−1^).

### Data analysis

Statistical significance of data obtained from Western blot, RT-PCR and electrophysiological recording was determined by unpaired Student's t-test with the use of Prism 5 (GraphPad Software, San Diego, CA). To determine the extent of Müller cells soma swelling, significance analysis was made using Mann-Whitney U-test for two groups and analysis of variance followed by comparisons for multiple groups.

## Supporting Information

Figure S1 Syntrophin expression after retinal detachment. Proteins were extracted from control and detached retina of C57BL/6 mice, 24 h after surgery. The protein samples were processed through SDS-PAGE and Western blotting, and probed with antibodies: anti-pan-syntrophin (SYN) and β-Actin. Monoclonal antibodies directed against syntrophins (α and β-SYN) and β-Actin were purchased respectively from Abcam (Cambridge, UK) and Sigma-Aldrich (Deisenhofen, Germany). The relative protein expression is expressed in arbitrary units as the mean + SE (n = 4). Each value represents the ratio of the specific band stain intensity normalized to β-Actin expression (TotalLab TL120, Nonlinear Inc, Durham NC, USA).
(0.09 MB TIF)

Figure S2 Immunolocalization of Dp71 in wt and Dp71-null retinal sections. In both mice, retinal sections were probed with a pan-specific antibody recognizing all dystrophin forms. In wt retinas, immunoreactivity with this antibody revealed a staining at the ILM (open arrowhead), around blood vessels (filled arrows), at the OPL (asterisk) and at the OLM (filled arrowhead). In Dp71-null retinas, solely the immunoreactivity at the OPL remains. The immunostaining at the OLM, around blood vessels and at the ILM disappears, confirming the Dp71 localization at these positions. OLM, outer limiting membrane; ONL, outer nuclear layer; OPL, outer plexiform layer; INL, inner nuclear layer; IPL, inner plexiform layer; GCL, ganglion cell layer; ILM, inner limiting membrane. Scale bar = 20 µm.
(0.06 MB PDF)
