# Supplementary figures and images for: Functional Implication of Dp71 in Osmoregulation and Vascular Permeability of the Retina
Source: PLoS One. 2009 Oct 7;4(10):e7329. doi: 10.1371/journal.pone.0007329 (PMC2754330; doi:10.1371/journal.pone.0007329)

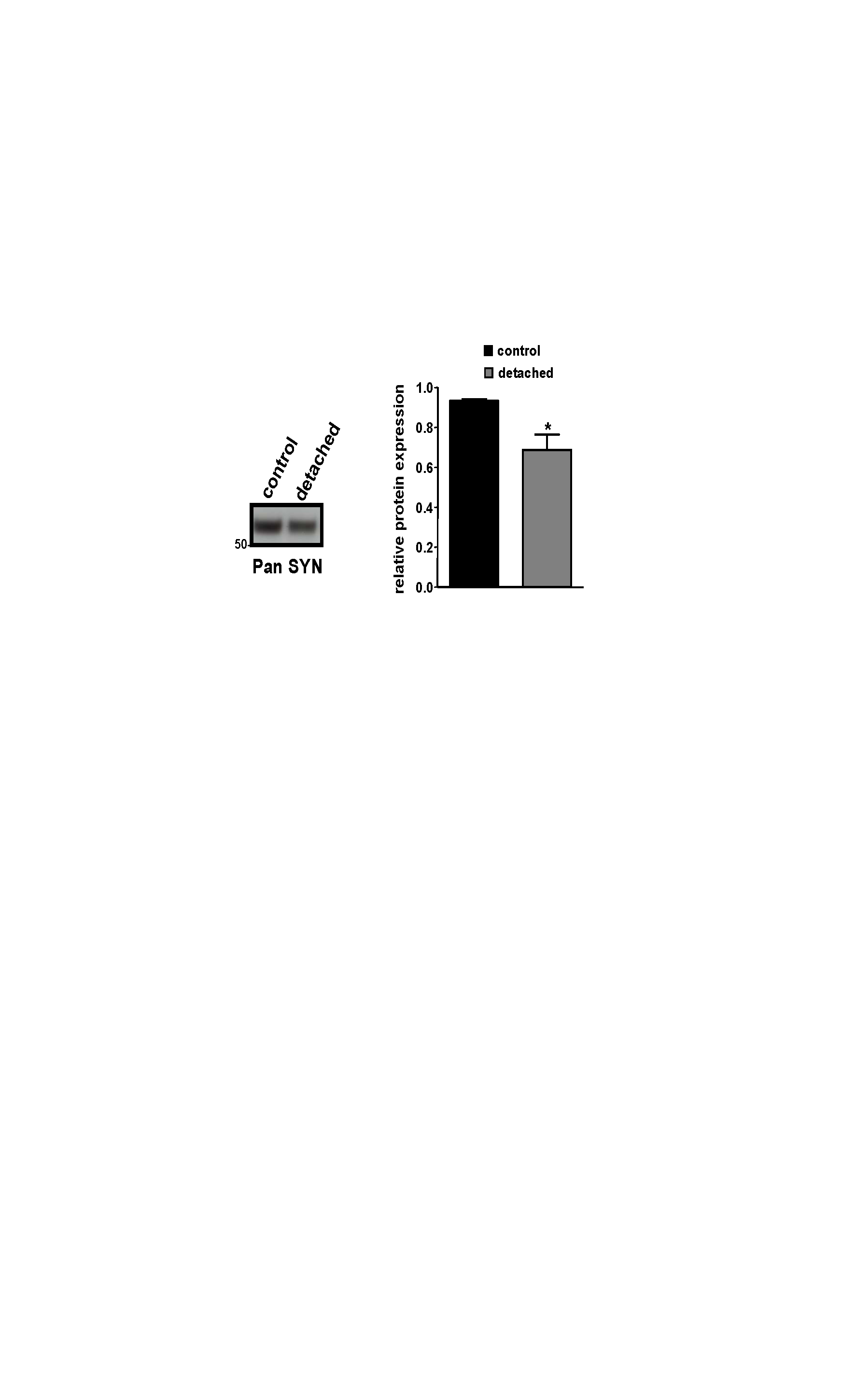

Supplement: Figure S1 — Syntrophin expression after retinal detachment. Proteins were extracted from control and detached retina of C57BL/6 mice, 24 h after surgery. The protein samples were processed through SDS-PAGE and Western blotting, and probed with antibodies: anti-pan-syntrophin (SYN) and β-Actin. Monoclonal antibodies directed against syntrophins (α and β-SYN) and β-Actin were purchased respectively from Abcam (Cambridge, UK) and Sigma-Aldrich (Deisenhofen, Germany). The relative protein expression is expressed in arbitrary units as the mean + SE (n = 4). Each value represents the ratio of the specific band stain intensity normalized to β-Actin expression (TotalLab TL120, Nonlinear Inc, Durham NC, USA). (0.09 MB TIF) [file pone.0007329.s001.tif]

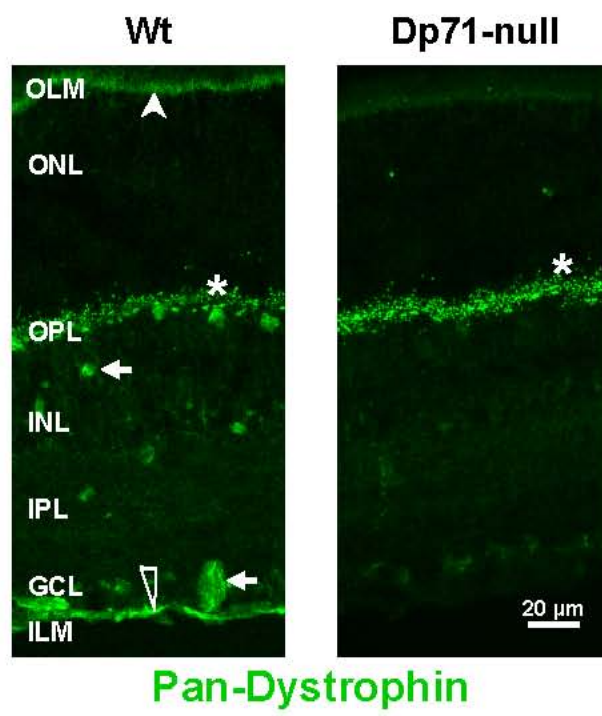

Supplement: Figure S2 — Immunolocalization of Dp71 in wt and Dp71-null retinal sections. In both mice, retinal sections were probed with a pan-specific antibody recognizing all dystrophin forms. In wt retinas, immunoreactivity with this antibody revealed a staining at the ILM (open arrowhead), around blood vessels (filled arrows), at the OPL (asterisk) and at the OLM (filled arrowhead). In Dp71-null retinas, solely the immunoreactivity at the OPL remains. The immunostaining at the OLM, around blood vessels and at the ILM disappears, confirming the Dp71 localization at these positions. OLM, outer limiting membrane; ONL, outer nuclear layer; OPL, outer plexiform layer; INL, inner nuclear layer; IPL, inner plexiform layer; GCL, ganglion cell layer; ILM, inner limiting membrane. Scale bar = 20 µm. (0.06 MB PDF) [file pone.0007329.s002.pdf]
